# Dorsolateral Prefrontal Cortex Glutamate/Gamma-Aminobutyric Acid (GABA) Alterations in Clinical High Risk and First-Episode Schizophrenia: A Preliminary 7-T Magnetic Resonance Spectroscopy Imaging Study

**DOI:** 10.3390/ijms232415846

**Published:** 2022-12-13

**Authors:** Ahmad Mayeli, Susan F. Sonnenschein, Victor E. Yushmanov, James D. Wilson, Annie Blazer, William Foran, Maria Perica, Finnegan J. Calabro, Beatriz Luna, Hoby P. Hetherington, Deepak K. Sarpal, Fabio Ferrarelli

**Affiliations:** 1 Department of Psychiatry, University of Pittsburgh, Pittsburgh, PA 15213, USA; 2 Department of Radiology, University of Pittsburgh, Pittsburgh, PA 15213, USA

**Keywords:** dorsolateral prefrontal cortex, DLPFC, first episode schizophrenia, gamma-aminobutyric acid, GABA, clinical high risk for psychosis, excitation/inhibition

## Abstract

Converging lines of evidence suggest that an imbalance between excitation and inhibition is present in the dorsolateral prefrontal cortex (DLPFC) of schizophrenia (SCZ). Gamma-aminobutyric-acid (GABA) and, to a lesser extent, glutamate (Glu) abnormalities were reported in the DLPFC of SCZ patients, especially on the right hemisphere, by post-mortem studies. However, in vivo evidence of GABA, Glu, and Glu/GABA DLPFC abnormalities, particularly on the right side and the early stages of illness, is limited. In this preliminary study, we utilized 7-Tesla magnetic resonance spectroscopic imaging (MRSI) to investigate bilateral Glu/Creatine (Cre), GABA/Cre, and Glu/GABA in the DLPFC of sixteen first episode schizophrenia (FES), seventeen clinical high risk (CHR), and twenty-six healthy comparison (HC) subjects. FES and CHR had abnormal GABA/Cre and Glu/GABA in the right DLPFC (rDLPFC) compared with HC participants, while no differences were observed in the left DLPFC (lDLPFC) among the three groups. Furthermore, HC had higher Glu/GABA in rDLPFC compared to lDLPFC (R > L), whereas the opposite relationship (R < L) was observed in the DLPFC Glu/GABA of FES patients. Altogether, these findings indicate that GABA/Cre and Glu/GABA DLPFC alterations are present before illness manifestation and worsen in FES patients, thus representing a putative early pathophysiological biomarker for SCZ and related psychotic disorders.

## 1. Introduction

Increasing evidence indicates that the dorsolateral prefrontal cortex (DLPFC) of schizophrenia (SCZ) is characterized by alterations in excitatory and inhibitory activity [1,2,3]. At the macroscopic level, neuroimaging studies have shown that patients with SCZ have reduced activation of DLPFC during tasks assessing working memory and cognitive control, both of which are impaired in these patients [4]. On a microscopic level, postmortem studies demonstrating morphological abnormalities in excitatory (i.e., glutamatergic pyramidal cells) and inhibitory (i.e., gamma-aminobutyric acid [GABA]-ergic interneurons) cells further suggest that the DLPFC is altered in SCZ and that these changes contribute to the pathophysiology of the disorder [5]. Specifically, several studies have reported abnormalities in GABA [6,7,8,9] and, less consistently, in glutamate (Glu) related markers [10,11] in the DLPFC of SCZ patients, including alterations in GABA-related transcriptomes and molecular markers of tonic inhibition, predominantly in the right hemisphere [6,7,8].

One approach for investigating these molecular abnormalities in SCZ in vivo involves using magnetic resonance spectroscopy (MRS), which allows quantifying DLPFC Glu and GABA. Most of the MRS work has focused on the left DLPFC (lDLPFC) of chronic patients with SCZ and found no differences in GABA or Glu levels in these patients compared to healthy comparison (HC) groups [12,13,14]. In contrast, there is a dearth of studies that investigated GABA and Glu alterations in bilateral DLPFC of SCZ patients [15]. Additional studies are especially needed in individuals experiencing the early stages of psychosis, including those at clinical high risk for psychosis (i.e., CHR), a critical time period with fewer confounds, such as prolonged medication exposure and chronicity, in order to: (a) implement early intervention strategies; (b) reduce the rate of transition to psychosis; and (c) decrease the risk of further progression of the disorder and impairment in everyday functioning [16]. Some recent MRS studies have reported unaltered GABA and Glu levels in the lDLPFC of first episode-schizophrenia (FES) patients [17,18]. However, none of these MRS studies have examined GABA and Glu in the rDLPFC of FES or CHR relative to HC subjects. 

Besides assessing both GABA and Glu in bilateral DLPFC in FES and CHR vs. HC, another important parameter to evaluate in these groups is the relative amount of these neurotransmitters (Glu/GABA), which is thought to reflect the DLPFC excitatory/inhibitory balance (E/I). Homeostatic control of the E/I balance is a requirement for efficient neuronal activity in the brain, which is maintained by a functional balance between the expression and activity of the glutamatergic and GABAergic systems [19]. Furthermore, impairments in cortical E/I balance have been reported in patients with SCZ and are thought to represent a core feature of the disorder [20,21]. Computational data have shown that cortical E/I imbalance, as reflected by altered Glu/GABA, may be critically implicated in the development and full manifestation of psychosis and SCZ [20,22]. Neurochemical and molecular biology studies have further examined Glu/GABA as a molecular marker of excitation/inhibition involved in the neuronal synthesis, degradation, and transport [23], key processes known to be altered in patients with SCZ [24]. Glu/GABA may therefore provide an early indication of abnormalities in SCZ [25]. Nonetheless, Glu/GABA has not been examined in the DLPFC of either FES or CHR individuals.

Employing magnetic resonance spectroscopic imaging (MRSI), also known as chemical shift imaging (CSI), enables spectral data to be acquired from multiple voxels within a single slice, multiple slices, or volumes simultaneously, providing information on the spatial distribution of GABA, Glu, and Glu/GABA [25,26,27]. Unlike single voxel measurements, where data is acquired from one location at a time, and each location requires a separate acquisition, MRSI methods accrue data from multiple locations (>100 pixels per slice) with approximately equivalent sensitivity per scan as single voxel spectroscopy (SVS) studies, thus offering a more efficient data collection when evaluating regionally specific changes. MRSI also enables cellular levels of GABA and Glu to be evaluated as potential readouts for GABAergic and glutamatergic activity. Relatedly, advances in high-field, 7-Tesla (7T) MRSI allow for high-resolution imaging across different brain structures. Compared to scans from lower field strengths, 7-Tesla MRSI provides greater spectral resolution, higher SNR, and improved detection and quantification of metabolites [28]. 

In this preliminary study, we employed 7T MRSI to measure Glu, GABA, and Glu/GABA in both left and right DLPFC in FES, CHR, and HC groups. Our overarching goal was to characterize abnormalities in these parameters in individuals at risk or in the early phases of SCZ, which in turn could contribute to identifying potential molecular pathophysiological biomarkers for psychotic disorders. 

## 2. Results

### 2.1. Clinical and Demographic Measures

Demographic variables and clinical measurements for each group of participants are provided in Table 1). Sixteen FES patients (four females, age [mean ± standard deviation]: 23.25 ± 4.88), seventeen CHR individuals (nine females, age: 19.98 ± 3.10), and twenty-six (fifteen females, age: 21.97 ± 4.57) HC subjects were included in the study. By applying one-way analysis of variance (ANOVA), we found no significant age differences across the 3 groups (*F(2,56)* = 2.452, *p* = 0.095). All our patients were recruited in accordance with the standard of care for psychotic disorders, which included blood work, urinalysis, and neurologic evaluations, when indicated, to rule out organic psychosis

### 2.2. Group Differences in DLPFC Glu/Creatine (Cre), GABA/Cre, and Glu/GABA

After confirming that the data were consistent with a normal distribution using Shapiro–Wilk normality test (*p* > 0.05; Table S1), we performed two-way analysis of covariances (ANCOVAs) for GABA/Cre, Glu/Cre, and Glu/GABA across the three groups (Table 2). For DLPFC GABA/Cre revealed significant differences for sex (*F(1,82)* = 5.27, *p* = 0.024), and group × hemisphere (*F(2,82)* = 4.72, *p* = 0.011, Table 2), whereas no significant effects of age, group, and hemisphere were found. In contrast, no significant differences were found for ANCOVA analysis for DLPFC Glu/Cre among age, sex, group, hemisphere, and group × hemisphere interaction (Table 2). Furthermore, ANCOVA analysis for DLPFC Glu/GABA revealed significant differences between sex (*F(1,83)* = 3.99, *p* = 0.049), and group × hemisphere (*F(2,83)* = 10.26, *p* < 0.001, Table 2). However, no significant effects of age, group, and hemisphere were established. 

Since we found a significant group by hemisphere interaction for GABA/Cre and Glu/GABA in DLPFC, for each of these parameters, we performed post hoc analyses to examine group differences in both hemispheres. 

**Figure 1:**
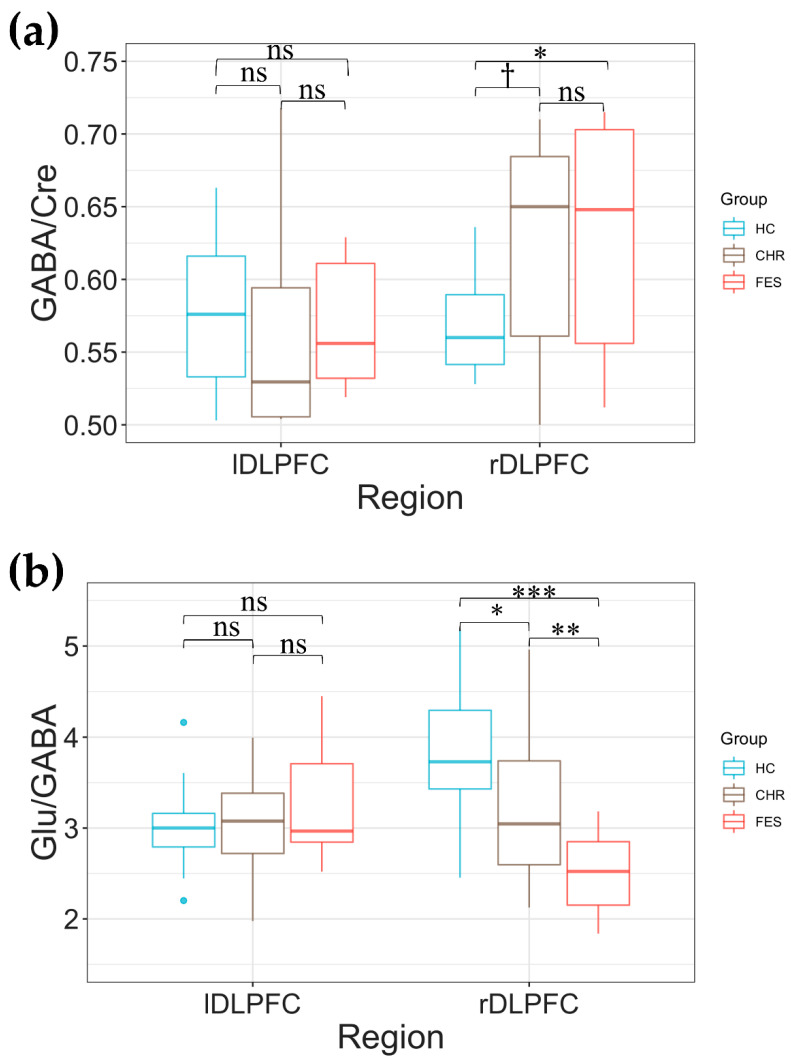
Box plots displaying the right and left dorsal lateral prefrontal cortex (rDLPFC and lDLPFC) GABA/Cre (**a**) and Glu/GABA (**b**) in healthy control (HC), clinical high risk (CHR), and first episode psychosis (FES) individuals. The *p* values are corrected for multiple comparisons using the false discovery rate method: ns represents non-significant differences, † *p* < 0.10, * *p* < 0.05, ** *p* < 0.01, and *** *p* < 0.001.

Post hoc analyses revealed higher rDLPFC GABA/Cre in FES (0.508 ± 0.115), and in CHR (0.477 ± 0.156) relative to HC (0.388 ± 0.104), which in FES vs. HC corresponded to false discovery rate (FDR)-corrected *p* < 0.011, *t-value* = 3.04, with a Cohen’s d effect size (*ES*) = 1.12 (Figure 1a), and in CHR vs. HC yielded a FDR-corrected *p* = 0.051, *t-value* = 2.18, with a Cohen’s d *ES* = 0.70 (Figure 1a). No differences were found in GABA/Cre between FES patients and CHR individuals (FDR-corrected *p* = 0.344, *t-value* = −0.96). Furthermore, no significant differences in lDLPFC Glu/GABA were observed between the 3 groups (FES vs. HC: FDR-corrected *p* = 0.73, *t-value* = −0.340, CHR vs. HC: FDR-corrected *p* = 0.62, *t-value* = −1.287, FES vs. CHR: FDR-corrected *p* = 0.65, *t-value* = 0.79). Further, no correlations were found between rDLPFC GABA/Cre and medication in FES (*p* = 0.280, *R* = 0.326). 

Regarding Glu/GABA, post hoc analyses showed that rDLPFC Glu/GABA was lower in both FES (2.485 ± 0.455) and CHR (3.272 ± 0.868) relative to HC (3.838 ± 0.708) which, in FES vs. HC, corresponded to FDR-corrected *p* < 0.001, *t-value* = −5.31, with a Cohen’s d *ES* = −2.12, and in CHR vs. HC to FDR-corrected *p* = 0.020, *t-value* = −2.40, and a Cohen’s d *ES* = −0.73 (Figure 1b). We also found that FES patients had lower rDLPFC Glu/GABA compared to CHR individuals that corresponded to an FDR-corrected *p* = 0.009, *t-value* = −2.86, and a Cohen’s d *ES* = −1.09 (Figure 1b). Of note, all rDLPFC Glu/GABA comparisons across groups yielded medium to large ESs. In contrast, no significant differences in Glu/GABA in lDLPFC were observed between the 3 groups (FES vs. HC: FDR-corrected *p* = 1.0, *t-value* = −0.004, CHR vs. HC: FDR-corrected *p* = 1.0, *t-value* = −0.08, FES vs. CHR: FDR-corrected *p* = 1.0, *t-value* = 0.07, Figure 1b). Moreover, no significant correlation between rDLPFC Glu/GABA level and medication in FES (*p* = 0.370, *R* = −0.274) was found. 

### 2.3. Glu/GABA Asymmetry in DLPFC across the Three Groups

**Figure 2:**
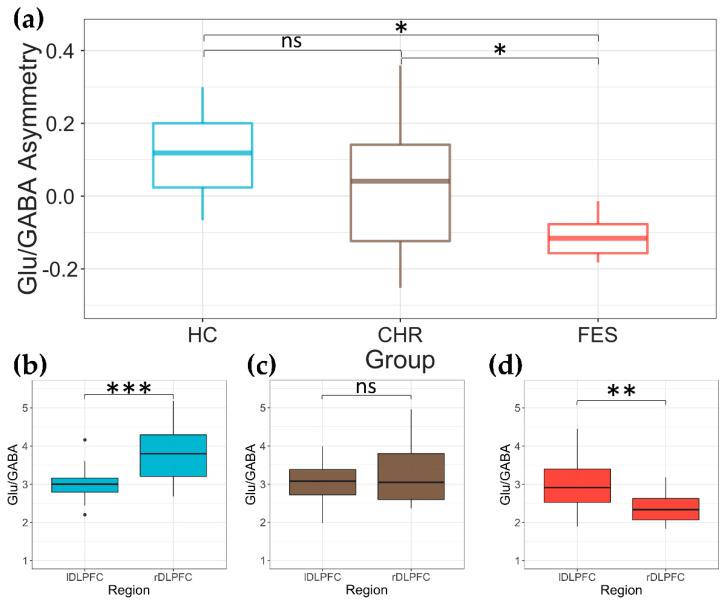
(**a**) The Glu/GABA asymmetry measure among the 3 study groups was computed as the difference between right and left DLPFC Glu/GABA over their summation. Box plots displaying the comparison between rDLPFC and lDLPFC Glu/GABA ratio in healthy control (HC, (**b**)), clinical high risk (CHR, (**c**)), and first episode schizophrenia (FES, (**d**)). We also computed paired *t*-test rDLPFC and lDLPFC Glu/GABA comparisons among groups; ns represents non-significant differences, * *p* < 0.05, ** *p* < 0.01, and *** *p* < 0.001.

We also assessed the relationship between Glu/GABA in the right and left DLPFC for each study group using an asymmetry measure (Equation (1); Figure 2a). The average (M) and standard deviation (STD) of the Glu/GABA asymmetry measure for each group were as follow (M ± STD): FES: −0.079 ± 0.115; CHR: 0.037 ± 0.211; HC: 0.101 ± 0.124. We found a significant difference for group (*F(2,32)* = 6.62, *p* = 0.004), whereas no significant effects of age (*F(1,32)* = 93, *p* = 0.343) and sex (*F(1,32)* = 0.23, *p* = 0.635) were established. Post hoc analysis revealed a significant asymmetry difference between FES and HC (FDR-corrected *p* = 0.030, t-value = −3.64, and Cohen’s d ES = −2.32) and FES vs. CHR (FDR-corrected *p* = 0.035, t-value = −2.38, and Cohen’s d ES = −0.97), while no difference between CHR and HC (FDR-corrected *p* = 0.214, t-value = −1.27, Figure 2a) were found. Furthermore, no correlations between medication and asymmetry in FES (*p* = 0.270, *R* = 0.44) were observed.

We also found that Glu/GABA in rDLPFC was significantly higher than lDLPFC (R > L) in HC individuals (paired *t*-test: *p* < 0.001, *t(16)* = 4.17, Cohen’s d *ES* = 1.01, Figure 2b, left panel), whereas the opposite relationship (R < L) was present in the DLPFC of FES patients (paired *t*-test: *p* = 003, *t(7)* = −4.46, Cohen’s d *ES* = 1.58, Figure 2d, right panel). There were no differences in Glu/GABA between right and left DLPFC in CHR participants (paired *t*-test: *p* = 0.41, *t(11)* = −0.86, Figure 2c, middle panel).

## 3. Discussion

In this study, we established GABA/Cre rDLPFC and Glu/GABA rDLPFC abnormalities in both FES patients and CHR individuals compared to HC subjects. FES and CHR had higher GABA/Cre and lower Glu/GABA relative to control participants. Moreover, while in HC participants, Glu/GABA in rDLPFC was higher than in the lDLPFC (R > L), no DLPFC Glu/GABA inter-hemispheric differences (L = R) were present in CHR individuals, and the opposite relationship (R < L) was observed in FES patients. It should be noted that all our FES patients had a diagnosis of schizophrenia except for one, who was diagnosed with schizoaffective disorder. Thus, it is highly unlikely that diagnostic heterogeneity affected our results.

The first important finding of the present study was that both CHR and FES individuals had elevated GABA/Cre levels compared to HC subjects. Post-mortem studies have shown abnormalities in GABAergic markers in SCZ, including a presynaptic reduction of GABA synthesis in SCZ patients compared to HC [6], a decrease in presynaptic GABA uptake [29], as well as an increase in postsynaptic GABA receptors [30,31] in SCZ patients compared to HC subjects. To account for these findings, it has been suggested that the initial pathologic process occurring in patients with SCZ is a presynaptic reduction of GABA synthesis, followed by a secondary, compensatory reduction of GABA presynaptic reuptake and upregulation of postsynaptic GABA receptor density/binding [32]. Alternatively, there could be an initial increase of GABAergic release due to both primary diminished reuptake and upregulated postsynaptic receptors, which in turn leads to a compensatory downregulation of presynaptic GABAergic activity [29]. Of note, due to difficulties in properly quantifying the net content of GABA, postmortem studies may not provide a conclusive answer about the net GABAergic concentrations and activity [29]. Additionally, post-mortem studies are conducted in chronic SCZ, and there may be differences in GABA levels between individuals in the early stages of psychosis and chronic patients because of possible confounds like duration of illness, medication exposure, and co-morbidities. In contrast, in vivo MRS studies can provide further insights into GABA levels in clinical states contributing to the pathological cascade and GABAergic alterations in schizophrenia, including early and at-risk stages. The present MRS findings appear to support an overall initial increase in GABA content in the rDLPFC, at least in at-risk and first episode SCZ patients. In contrast, no alterations in GABA/Cre in FES or CHR relative to HC in the lDPLFC. Previous in vivo spectroscopy studies investigated GABA and Glu level abnormalities in the left DLPFC of CHR and SCZ and reported no alterations in GABA or Glu levels in these clinical populations compared to HC subjects [33,34,35,36]. Our findings are, therefore, consistent with this work. An important research implication of this finding is that rDLPFC may be better suited than lDLPFC to capture GABA alterations, especially in at-risk and early phases of SCZ, and therefore future studies should be focused on this area. 

We also found no differences in Glu/Cre across the three groups for the right and left DLPFC. Previous MRS work assessing Glu in bilateral DLPFC reported conflicting findings. One study found a significant reduction in Glu level in chronic SCZ patients compared to HC [37], while another study reported an elevated Glu level in first episode psychosis patients relative to their HC subjects [38]. Overall, most of the MRS studies found no difference in DLPFC Glu levels between SCZ patients, as recently established by a meta-analysis [39]. Similarly, a couple of recent MRS studies examining glutamate levels in CHR [40,41] reported no differences between cases and controls, which is consistent with the negative findings reported here. 

A decrease in rDLPFC Glu/GABA in both CHR individuals and FES patients compared to HC subjects was also observed in this study. The balance between excitation and inhibition (E/I) is a classic approach for modeling neural activities and brain function [42,43] and can be measured by the relative amount of Glu and GABA, which represent the main excitatory and inhibitory neurotransmitters in the brain, as Glu/GABA [44]. An E/I imbalance has been rarely assessed in pre-clinical and clinical studies. Specifically, a recent 9.4T MRS study reported a decrease in Glu/GABA in the prefrontal cortex of Cntnap^2−/−^ mice, which has been implicated in autism spectrum disorder, compared to their wild-type littermates [45], while we recently reported a decrease in GABA/Glu in the thalamus of CHR vs. HC [25]. The decrease in prefrontal Glu/GABA in both at-risk and first episode SCZ relative to HC found here indicates that these abnormalities are present in the early phases of psychosis and can be detected even before the manifestation of the disorder. We also determined that FES patients had significantly lower Glu/GABA levels compared to CHR participants, suggesting a worsening across clinical stages of illness, from prodromal to first episode SCZ. This finding could have relevant implications both in the research and clinical endeavors. Concerning the former, Glu/GABA may represent a parameter that could be utilized to further characterize the pathophysiology of SCZ, including at risk and early stages, across several methodological approaches, ranging from *post-mortem* to in vitro, from in vivo to in silico. Regarding the latter, we can speculate that alterations in rDLPFC Glu/GABA, which are present in at-risk individuals, may be utilized as a predictive biomarker in the prodromal phases of the disorder as well as a monitoring biomarker to assess disease progression following the first episode of SCZ. 

We also found that Glu/GABA was significantly higher in rDLPFC relative to lDLPFC in HC subjects. This R > L asymmetry was lost in CHR participants, who showed similar Glu/GABA between ldPFC and rDLPFC. In contrast, in FES patients, this asymmetry was inverted, with R < L prefrontal Glu/GABA. Although there is a dearth of work investigating DLPFC hemispheric asymmetry in excitatory and inhibitory balance in both HC and SCZ individuals, a recent study employing a dual-coil paired-pulse Transcranial Magnetic Stimulation paradigm demonstrated that lDLPFC has a greater inhibitory effect compared to the rDLPFC in HC subjects [46], which is in line with lower lDLPFC Glu/GABA in HC. We were able to establish these alterations in DLPFC asymmetry in CHR and FES individuals relative to HC subjects by simultaneously assessing Glu/GABA in the left and right DLPFC with 7T MRSI. Specifically, MRSI, which allows us to assess multiple-voxel locations (i.e., right, and left DLPFC) at the same time, can measure different spectra in parallel and provides a better signal to noise ratio (SNR) for multiple regions of interest, while higher filed strength (i.e., 7T) heightens the SNR and narrows the peak and narrow peak widths. This results in improved spectral resolution and sensitivity, which in turn enables a more accurate quantification of neurotransmitters and the detection of overlapping metabolites (i.e., macromolecules and GABA) [47]. 7T MRSI can therefore be used to answer important research questions, including the specificity and asymmetry of Glu/GABA alterations in homologous brain regions beyond the DLPFC, which may eventually lead to more targeted interventions in patients affected by SCZ at different stages of the disorder. 

Future work is needed to address some of the questions left unanswered here. First, in this preliminary study, the sample size of FES and CHR was relatively small. Both CHR and FES are populations that are difficult to recruit. Furthermore, most of this study happened during the COVID pandemic, which also negatively affected the recruiting process. The presented results, therefore, are preliminary and provide directions for future investigations that are needed to replicate these findings in larger groups of at-risk and FES patients. Nonetheless, the DLPFC GABA and Glu/GABA alterations observed here yielded medium to large effect sizes in FES and CHR patients relative to HC. Second, while we aimed at recruiting age-matched groups, CHR individuals tended to be younger than FES since the at-risk phase proceeds the development of full-blown psychosis. Nevertheless, there was no significant age difference across study groups. Furthermore, to account for the possible effect of age on the main findings, we used age as a covariate for all analyses. Relatedly, although the three groups were not matched by gender, an issue that should be addressed in future studies, we utilized sex as a covariate for all the statistical analyses and found that this variable did not significantly affect our main findings. Third, the MRSI findings reported here are cross-sectional. Longitudinal studies are therefore needed to assess whether decreased Glu/GABA in rDLPFC and bilateral DLPFC asymmetry can predict and/or track illness trajectory in different stages of psychosis. However, the DLPFC alterations that we established in the present study were progressively worse from at risk to first episode SCZ, consistent with a putative molecular monitoring biomarker of disorder progression. Fourth, the DLPFC plays a crucial role in regulating working memory (WM) and related cognitive functions, including executive [48], verbal WM [49], and visual-spatial WM [50,51]. Future studies in FES and CHR should therefore investigate the relationship between cognition and GABA/Cre, Glu/Cre, and Glu/Cre. Fifth, in this study, we focused on investigating DLPFC alterations. Building on these findings, future work should examine possible GABA and Glu/GABA alterations in other cortical and subcortical regions. For instance, in a recent study, we reported altered GABA and GABA/Glu in the thalamus of CHR relative to HC [25]. Finally, future studies should examine differences in other neurotransmitters and see how they relate to GABA and Glu parameters in those clinical groups. Nonetheless, the present study provided the first preliminary in vivo molecular evidence of DLPFC excitatory/inhibitory imbalance in at-risk and early stages SCZ patients.

## 4. Materials and Methods

### 4.1. Participants

Sixteen FES patients, seventeen CHR individuals, and twenty-six HC subjects were recruited for this study. 

### 4.2. Recruitment, Eligibility Criteria, and Clinical and Cognition Measurements

The study was conducted at the University of Pittsburgh and approved by the University of Pittsburgh Institutional Review Board. All participants over 18 years provided written informed consent prior to completing study procedures; in case the participant was under 18 years, informed consent was obtained from parents. Participants received financial compensation for participation in the study. To avoid any coercion into participating in our study, when consenting individuals, we clarified that their participation was voluntary and that they could terminate their involvement in the study at any point.

HC individuals were recruited from the local community through online and physical advertisements. CHR and FES individuals were also enrolled through UPMC clinical settings, referrals from other clinicians, and outside sources. 

General eligibility criteria for potential participants included: (1) ages between 12 and 35 for HC and CHR and between 18 and 40 years for FES; (2) no lifetime history of a diagnosed neurological disorder or head injury resulting in loss of consciousness for more than one minute; (3) being able to speak English fluently enough to participate in clinical assessments and study procedures; (4) being able to travel to Western Psychiatric Hospital to participate in the assessments; (5) no pregnancy; (6) no history of alcohol or drug dependence in the past 12 months; and (7) ability to provide informed consent. Additional exclusion criteria for HC participants were: (1) Axis-1 disorder diagnosis, assessed with the structured clinical interview for DSM-IV disorders (SCID-IV); (2) high-risk syndrome diagnosis; (3) first-degree relatives with a known psychotic disorder diagnosis. 

Additional eligibility criteria for FES groups were: (1) patients in the early phase of current DSM-defined diagnosis of schizophrenia, schizophreniform disorder, and schizoaffective disorder; (2) antipsychotic medication use for a cumulative lifetime period of ≤1 year; and (3) prior to or within 1 week of first antipsychotic drug initiation by the clinical team. Organic psychoses were excluded per routine specialized clinical care services at UPMC Western Psychiatric Hospital that are dedicated to serious mental illness and psychotic disorders. Specifically, our patients were recruited from clinical services with expert evaluations in accordance with the standard of care for psychotic illness, which includes blood work, urinalysis, and neurologic evaluations, when indicated, to rule out organic psychosis. Furthermore, neuropsychological assessments and MRI were performed in all patients enrolled in this study.

We utilized DSM-IV criteria to diagnose schizophrenia because the study began before the DSM-5 was operationalized in our research group. Specifically, our study procedures derived from existing protocols and validated methods for recruitment and assessment that were initiated before the adoption of DSM-5 diagnoses. Furthermore, there have been only minimal changes in the diagnostic criteria for schizophrenia between DSM-IV and DSM-5 [52].

### 4.3. ^1^H MRSI Data Acquisition and Processing

MRSI data were acquired on a 7 T Siemens Magnetom scanner using an 8-channel multiple transmit system with a body gradient coil and an 8 × 2 transceiver array (2 rows, 8 coils/row, Resonance Research Inc.) A Very High Order Shim (VHOS) insert coil (with a 38 cm inner diameter) providing 3rd, 4th order shims and partial 5th order shims (Resonance Research Inc.) was used for higher degree/order *B*_0_ shimming [53] in conjunction with the standard 1st and 2nd order corrections available from the gradient coil. The VHOS reduces in-plane magnetic field inhomogeneity by ~50% in comparison to that achievable using only 1st & 2nd order shims [53]. This improvement enhances spectral quality throughout the slice, enabling more accurate measurements of GABA and Glu [54,55]. The large molar lipid signals from outside of the brain were suppressed using *B*_1_ shimming-based outer volume suppression. Thus, two *B*_1_^+^ distributions were used: a ‘homogeneous’ distribution for excitation of the brain and a ‘ring’ distribution to selectively suppress extra-cranial tissues, as described previously [56]. 

Planar MRSI data were acquired in a slice angulated along the DLPFC plane using a slice selective J-refocused coherence transfer sequence [26] with TE/TR = 34/1500 ms, matrix size = 24 × 24 over a FOV of 216 mm × 216 mm, and slice thickness = 10 mm (nominal voxel size = 9 mm × 9 mm × 10 mm). The J-refocused sequence suppresses the J-modulation of GABA and Glu at moderate TEs, preserving signal-to-noise-ration (SNR) and spatial resolution and reducing broad baseline components, thereby improving the measurement accuracy [26,57]. Four Hz of Gaussian broadening was used in the spectral domain along with a 100-Hz convolution difference. A Hanning filter was applied in the spatial domain prior to Fourier transformation resulting in in-plane distribution having a full width at half maximum diameter of ~16.9 mm. The integrated volume weighted by efficiency is identical to an unfiltered data set. Water suppression was achieved via a frequency-selective inversion recovery preparation module and optimized semi-selective refocusing pulses [56]. An MP2RAGE sequence [58] with TR of 6000 ms and TI of 800 and 2700 ms provided 1.0 mm isotropic resolution in a FOV of 240 × 240 × 192 mm for anatomic identification. Scout images contained 33 3-mm-thick 128 mm × 128 mm slices (in-plane resolution of 1.7 mm). The matched scout images were used to weigh and phase the data for coil recombination.

FreeSurfer (Massachusetts General Hospital, Harvard Medical School; http://surfer.nmr.mgh.harvard.edu, accessed on 20 January 2022) was used to segment the anatomical brain image (MP2RAGE) into different cortical regions of interest. Statistical Parametric Mapping (SPM12; http://www.fil.ion.ucl.ac.uk/spm, accessed on 20 December 2021) was used to co-register the scout image to the MP2RAGE and further transform MP2RAGE into the MNI space to assess the voxel center MNI coordinates and assign voxels to Brodmann areas.

LCModel was utilized to quantify the spectral data (in the range of 1.8 to 4.0 ppm) [59], which consist of default macromolecule components and 14 basis metabolite functions (N-acetylaspartate (NAA), N-acetylaspartylglutamate (NAAG), aspartate, lactate, creatine (Cre), γ-aminobutyric acid (GABA), glucose, glutamate (Glu), glutamine, glutathione, glycerophosphorylcholine (GPC), phosphorylcholine (PCh), myoinositol, and taurine). The basis functions were calculated from GAMMA simulations incorporating the effects of the pulse sequence [60]. To avoid potential quantification errors introduced by the partial volume effects through the inclusion of cerebrospinal fluid, transmit inhomogeneity due to high field effects and spatially varying sensitivity due to the use of a receive array, metabolite ratios (by using Cre as the denominator in metabolite ratios), rather than their absolute concentrations, were calculated. Cre is often used as an internal reference for other metabolites due to being a metabolite with a strong signal, reliable chemical shift, and good stability, in addition to allow for a shorter acquisition time compared to using a water reference. Six neurometabolite ratios: tNAA/Cre, GABA/Cre, Glu/Cre, tCho/Cre, Gln/Cre, and Glu/GABA, were computed, with Cre representing the sum of creatine and phosphocreatine, tNAA the total of NAA and N-acetylaspartylglutamate (NAAG), and tCho the total of major choline-containing compounds, glycerophosphorylcholine (GPC) and phosphorylcholine (PCh)). For the present study, we focused on the relative concentration of GABA and Glu (i.e., Glu/GABA) in the right and left DLPFC as a measure of GABA-glutamate balance. Thus, Glu/GABA was computed as $\frac{\frac{Glu}{Cre}}{\frac{GABA}{Cre}}$. 

**Figure 3:**
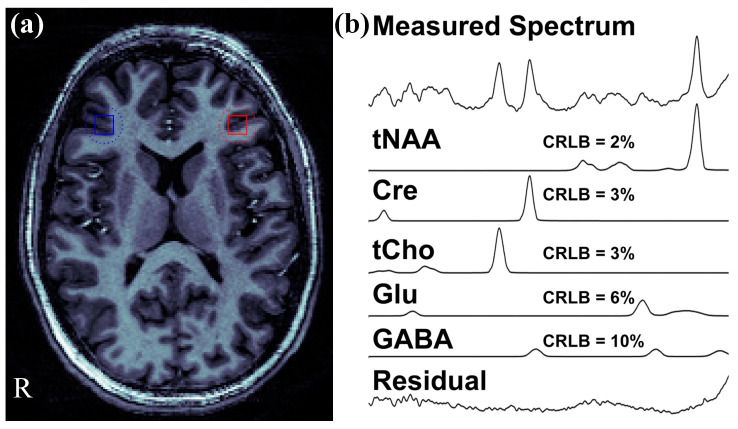
(**a**) Representative location for the right dorsolateral prefrontal cortex (DLPFC) (blue square) and the left DLPFC (red square) of a first episode schizophrenia (FES) patient. (**b**) Individual fits for the major compounds of interest, their Cramer–Rao lower bound (CRLB) values and a residual (a difference between the measured and fitted spectra) of magnetic resonance spectroscopic imaging (MRSI) spectrum of the right DLPFC region of interest (presented in part **a**).

The LCModel provides Cramer-Rao Lower Bounds (CRLB) values for each neurotransmitter as an expression of the estimate’s uncertainty for any metabolites. More specifically, the CRLB is a measure of the data quality in which it estimates the lowest possible variance for an estimator and indicates how well the measurement of a given metabolite fits the ideal spectrum for that metabolite. The CRLB values for each ratio were utilized to filter out data of poor spectral quality. Spectra were excluded if CRLB > 10 for the major singlet resonances tNAA, Cre, or tCho, or CRLB > 20 for GABA and/or Glu. For each participant, we selected ROIs for the rDLPFC and lDLPFC from the image that was co-registered into MNI and confirmed the placement that these ROIs were in Brodmann area (BA) 9 or BA 46. Figure 3a represents the location of selected brain regions (i.e., right and left DLPFC). These coordinates were then used in a voxel-shifting reconstruction [61] to center the ROI location with 1 mm accuracy. Thus, partial volume effects due to prescan positioning of the MRSI grid were minimized. The resulting data was then analyzed with LCModel to obtain the final values for each compound. A representative spectrum of an FES patient is shown in Figure 3b.

In addition to measuring the Glu/GABA in DLPFC, we compared right and left DLPFC Glu/GABA values to assess for between-hemisphere asymmetry (e.g., lDLPFC Glu/GABA higher than rDLPFC Glu/GABA) using a paired *t*-test among each group of subjects. To quantify this difference, we defined an asymmetry measure as follows:

(1) $$Asymmetry=\frac{rDLPFC\frac{Glu}{GABA}-lDLPFC\frac{Glu}{GABA}\;}{rDLPFC\frac{Glu}{GABA}+lDLPFC\frac{Glu}{GABA}\;}$$

### 4.4. Statistical Analysis

We used Shapiro–Wilk test for assessing the normality of all parameters and after confirming that the data were consistent with a normal distribution, we utilized parametric tests for any further analysis. A two-way analysis of covariance (ANCOVA) with hemisphere and group as independent variables, a neurotransmitter level as the dependent variable, as well as age and sex as covariates was conducted for each neurotransmitter ratio (i.e., GABA/Cre, Glu/Cre, Glu/GABA). We applied pairwise comparisons across three study groups when the ANCOVA global F test showed significant differences across groups. In order to correct for multiple comparisons, we utilized the false discovery rate (FDR) method [62,63]. A paired *t*-test was applied to compare right and left DLPFC levels between each participant group separately. Cohen’s effect size (ES) was calculated for all significant paired and unpaired *t*-tests [64]. 

## 5. Conclusions

In summary, in this preliminary study, by using 7 T MRSI in CHR individuals, FES patients, and HC subjects we established that GABA-driven Glu/GABA DLPFC alterations are present before illness onset, worsen in first-episode patients, and are associated with their cognitive deficits, thus representing a putative early pathophysiological biomarker for SCZ and related psychotic disorders.

## Figures and Tables

**Table 1:** Clinical and demographic measures of the study participants.

Measures	Healthy Control	Clinical High Risk	First Episode Schizophrenia	*p*-Value
Number of subjects	26	17	16	-
Sex (# female)	15	9	4	-
Average Age ± SD in years (range)	21.97 ± 4.57 (14–33)	19.98 ± 3.10 (15–26)	23.25 ± 4.88 (18–33)	0.095 ^1^
SOPS_PS (average ± SD)	-	12.875 ± 2.83	-	-
SOPS_NS (average ± SD)	-	12.56 ± 3.81	-	-
SOPS_DS (average ± SD)	-	5.19 ± 1.64	-	-
SOPS_GS (average ± SD)	-	7.00 ± 2.87	-	-
BPRS (Total)	-	-	53.56 ± 9.79	-
BPRS (Negative Symptoms)	-	-	8.62 ± 3.09	-
BPRS (Hallucination Symptoms)	-	-	13.56 ± 3.58	-
BPRS (Positive Symptoms)	-	-	16.50 ± 3.76	-

^1^ Analysis of variance (ANOVA) *p*-value.

**Table 2:** Two-way analysis of covariances (ANCOVA) results for Glu/Cre, GABA/Cre, and Glu/GABA.

		DLPFC		
		Glu/Cre	GABA/Cre	Glu/GABA
Group	DF	2	2	2
	F-Value	1.151	0.948	0.167
	*p*-Value	0.321	0.392	0.846
Hemisphere	DF	1	1	1
	F-Value	1.383	0.083	1.128
	*p*-Value	0.243	0.773	0.291
Group × Hemisphere	DF	2	2	2
	F-Value	0.96	4.723	10.26
	*p*-Value	0.387	**0.011**	**<0.001**
Age	DF	1	1	1
	F-Value	0.0004	0.14	0.544
	*p*-Value	0.984	0.709	0.463
Sex	DF	1	1	1
	F-Value	0.141	5.266	3.993
	*p*-Value	0.708	**0.024**	**0.049**

Glu: glutamate; GABA: Gamma-aminobutyric acid; Cre: Creatine; DF: degree of freedom.

## Data Availability

Data can be requested from the authors.

## Supplementary Materials

The following supporting information can be download at: https://www.mdpi.com/article/10.3390/ijms232415846/s1, Table S1. Shapiro-Wilk normality test results for all parameters.
