# Supplementary material for: Dorsolateral Prefrontal Cortex Glutamate/Gamma-Aminobutyric Acid (GABA) Alterations in Clinical High Risk and First-Episode Schizophrenia: A Preliminary 7-T Magnetic Resonance Spectroscopy Imaging Study
Source: Int J Mol Sci. 2022 Dec 13;23(24):15846. doi: 10.3390/ijms232415846 (PMC9781166; doi:10.3390/ijms232415846)
Supplement: Supplementary file 1 [file ijms-23-15846-s001.zip › ijms-1986668 - supplementary.pdf]

International Journal of *Molecular Sciences*

MDPI

Supplementary Materials

Dorsolateral Prefrontal Cortex Glutamate/Gamma-Aminobutyric Acid (GABA) Alterations in Clinical High Risk and First-Episode Schizophrenia: A Preliminary 7-T Magnetic Resonance Spectroscopy Imaging Study

Ahmad Mayeli [1,*], Susan F. Sonnenschein [1], Victor E. Yushmanov [2], James D. Wilson [1], Annie Blazer [1], William Foran [1], Maria Perica [1], Finnegan J. Calabro [1], Beatriz Luna [1], Hoby P. Hetherington [2], Deepak K. Sarpal [1] and Fabio Ferrarelli [1,*]

[1] Department of Psychiatry, University of Pittsburgh, Pittsburgh, PA 15213, USA
[2] Department of Radiology, University of Pittsburgh, Pittsburgh, PA 15213, USA
* Correspondence: mayelia@upmc.edu (A.M.); ferrarellif@upmc.edu (F.F.)

Table S1. Shapiro-Wilk normality test results for all parameters.

Parameter	ROI	Group	W	*p*-Value
Glu/GABA	DLPFC	HC	0.965	0.231
		CHR	0.960	0.344
		FES	0.953	0.357
	rDLPFC	HC	0.971	0.667
		CHR	0.934	0.284
		FES	0.946	0.577
	lDLPFC	HC	0.962	0.667
		CHR	0.958	0.757
		FES	0.910	0.315
Glu/Cre	DLPFC	HC	0.958	0.125
		CHR	0.960	0.345
		FES	0.981	0.218
	rDLPFC	HC	0.942	0.183
		CHR	0.956	0.619
		FES	0.953	0.641
	lDLPFC	HC	0.970	0.797
		CHR	0.929	0.327
		FES	0.887	0.185
GABA/Cre	DLPFC	HC	0.956	0.114
		CHR	0.964	0.453
		FES	0.953	0.357
	rDLPFC	HC	0.935	0.125
		CHR	0.956	0.583
		FES	0.886	0.086
	lDLPFC	HC	0.940	0.316
		CHR	0.942	0.549
		FES	0.949	0.675
Glu/GABA asymmetry	DLPFC	HC	0.944	0.367
		CHR	0.953	0.679
		FES	0.910	0.351
